# Features and amenities of school playgrounds: A direct observation study of utilization and physical activity levels outside of school time

**DOI:** 10.1186/1479-5868-8-32

**Published:** 2011-04-14

**Authors:** Natalie Colabianchi, Andréa L Maslow, Kamala Swayampakala

**Affiliations:** 1 Department of Epidemiology and Biostatistics, Arnold School of Public Health, University of School Carolina, 800 Sumter Street, Columbia, SC 29208, USA; 2 R. Stuart Dickson Institute for Health Studies, Carolinas HealthCare System, PO Box 32861, Charlotte, NC, 28232, USA; 3 Public Health Consortium, 800 Sumter Street, Columbia SC 29208, USA

## Abstract

**Background:**

A significant amount of research has examined whether park or playground availability is associated with physical activity. However, little research has examined whether specific features or amenities of parks or playgrounds, such as the number of unique types of playground equipment or the safety of the equipment is associated with utilization of the facility or physical activity levels while at the facility. There are no studies that use direct observation and a detailed park assessment to examine these associations.

**Methods:**

Twenty urban schoolyards in the Midwest, ten of which were renovated, were included in this study. Using a detailed environmental assessment tool (i.e., Environmental Assessment of Public Recreation Spaces), information on a variety of playground attributes was collected. Using direct observation (i.e., System for Observing Play and Leisure Activity in Youth), the number of adults, girls and boys attending each schoolyard and their physical activity levels were recorded. Each schoolyard was observed ten times for 90 minutes each time outside of school hours. Clustered multivariable negative binomial regressions and linear regressions were completed to examine the association between playground attributes and utilization of the schoolyard and the proportion active on the playground, respectively. Effect modification by renovation status was also examined.

**Results:**

At renovated schoolyards, the total number of play features was significantly associated with greater utilization in adults and girls; overall cleanliness was significantly associated with less utilization in girls and boys; and coverage/shade for resting features was significantly associated with greater utilization in adults and boys. At unrenovated schoolyards, overall safety was significantly associated with greater utilization in boys. No playground attribute was associated with the proportion active on the playground after adjusting for all other significant playground attributes.

**Conclusions:**

Having a large quantity of play features and shade at renovated playgrounds were positively associated with utilization of the schoolyard. Modifying playgrounds to have these features may increase the utilization of these facilities outside of school time. Additional research should explore what features and amenities are associated with increased physical activity levels of children and adults who utilize the facilities.

## Background

In an effort to increase physical activity levels, recent research has focused on the built environment. An abundance of research has suggested that park or playground availability is associated with increased activity for children [1-3] and adults [4-7]. However, few of these studies have examined whether specific features of parks or playgrounds such as number of elements on a playground or quality of features are associated with greater utilization or increased activity levels of those using these facilities [4,8,9].

Studies in adults that have examined features of parks or playgrounds have generally examined broad aspects such as the availability of a walking trail or a basketball court. For example, Shores and West examined 6 features of four parks including availability of courts, green space, paths, playgrounds, sports fields, or shelter/picnic areas and correlated these features with observed activity levels of visitors [10]. They found four features were associated with activity levels of park users including playgrounds, courts and paths (all positively associated) and shelters (negatively associated). Another study of two US cities found activity levels measured by direct observation varied across target areas, which largely corresponded to recreation use [6]. Assessed target areas included courts (i.e. tennis, racquetball, volleyball, and basketball), sports fields (i.e. soccer, football, and baseball/softball), shelters, playgrounds, and open spaces. They reported the greatest amount of energy expenditure was at tennis/racquetball courts and basketball courts while the lowest amount was reported at picnic shelters. In a similar study, Reed et al examined 25 parks to determine the most and least frequently used activity settings using direct observation [11]. They reported paved trails were the most frequently used activity setting for both genders, while softball and baseball fields were the second most heavily used activity setting for males and swimming pools were the second most commonly used setting for females.

Only one study of adults used a detailed assessment of park features and amenities, which was correlated with self reported physical activity [12]. They found that the availability of more features (e.g., trails, wooded area, playground, base diamond, basketball court), in particular trails, was associated with adult utilization of the parks for physical activity. Number of amenities (i.e., drinking fountains, benches, trash cans) was not associated with park utilization for physical activity after controlling for number of features.

A few studies have examined whether proximity to parks with specific features was associated with physical activity levels in children and adolescents, though none of these studies captured whether the participants gained activity at the park locations. Specifically, Cohen et al found that girls who lived near parks with running tracks, playgrounds, basketball courts, street and flood lights were more likely to have higher levels of vigorous activity outside of school as measured by accelerometry relative to girls who did not have parks with these features and amenities nearby [13]. Another study found the availability of a playground within one kilometer of a child's home was associated with a healthy weight in children as self reported by the parent [2]. In this same study, availability of 12 other park features such as paved trails, ball diamonds and basketball courts were not associated with healthy weight status in children. Finally, Timperio, Giles-Corti, Crawford et al., examined 10 features of the nearest public open space (e.g., availability of paths, lighting, signage, number of playgrounds) with physical activity levels as measured by accelerometry after school and on weekends in children 8-9 years and 13-15 years old [14]. Inconsistent results across age and gender were found in this study.

Two studies have looked at physical activity levels and features of playgrounds or schoolyards specifically. Haug, Torsheim, Sallis and Samdal found that, across 130 schools in Norway, secondary students (grades 8-10) self reported 2.5 times more vigorous active during recess when they attended schools with a larger number of outdoor facilities as reported by the principal [15]. No association was seen for those in primary school (grades 4-7). In a study of preschool playgrounds, Cardon and colleagues found higher step counts, as measured by a pedometer, were associated with less children per square meter at the playground [16]. For boys, having a hard surface was a borderline significant predictor of step counts and for girls having fewer supervising teachers was associated with fewer step counts in unadjusted associations. Neither study collected data on which areas of the schoolyard the physical activity was occurring in.

In addition to observational studies, a few studies have documented the effects of playground renovations on physical activity levels and/or utilization. Researchers in the UK found that painting multicolor playground markings and providing limited physical structures (e.g., soccer goal posts, basketball hoops) resulted in greater physical activity levels in both the short term [17] and longer - term (i.e., 6 months) compared to control schools [18]. Other playground renovations studies have found increased utilization at renovated schoolyards and greater physical activity levels, particularly in boys [19,20].

In sum, there is little research examining whether specific features or amenities of parks or playgrounds, such as the number of unique types of playground equipment or the quality of the features are associated with utilization of the facility or physical activity levels in children and adults once they are there. No studies were identified that used direct observation and a detailed park assessment to examine these associations. This study attempted to address this gap by examining attributes of 20 school playgrounds and their association with the number of persons at the schoolyard outside of school time (hereafter referred to as utilization) and physical activity levels of those on the playground (hereafter referred to as physical activity level).

## Methods

Twenty urban elementary schoolyards in Cleveland, OH were included in the study. These schoolyards were selected as part of an evaluation study of schoolyard renovations [20]. As part of this renovation, titled School Grounds as Community Parks, schoolyards received new playground equipment and safety and site improvements. Ten schools that had been renovated for at least a year were included along with ten unrenovated schools. All schools had functional playgrounds regardless of renovation status and were neighborhood schools, meaning those who attended the school lived in the surrounding area (i.e., non-magnet school).

Utilization and activity levels of the users were measured by direct observation using the System for Observing Play and Leisure Activity in Youth (SOPLAY) [21]. Each schoolyard was observed on ten occasions outside of school time spanning evening (4:30-6:00 pm or 6:30-8:00 pm) and weekend times (10:30-12:00 am, 12:30-2:00 pm, or 2:30-4:00 pm). Features of the playground were documented using the Environmental Assessment of Public Recreation Spaces (EAPRS) [22] one month prior to the direct observations, which took place May-July, 2005.

At the time of the study, the city's 414,500 residents were comprised of 54% African American residents, 39% White residents, 2% Asian residents and 5% residents of other races. Just under 8% of residents classified themselves as Hispanic, regardless of race. In the census tracts of the schools included in this study, an average 39% of the children under 17 lived in poverty. The participants of this study are those who came to the schoolyards during the observation periods. This protocol was reviewed by the MetroHealth Institutional Review Board and deemed exempt.

### Exposures

Portions of the EAPRS measurement tool that focused on the playground features and amenities were completed at each of the schoolyards including the following sections: play set and structure features, other components (separate from play set), general areas, eating/drinking features, sitting or resting features (non-trail), general aesthetics, directives and information-related features. The first author was trained on the instrument by the developer of the EAPRS. The first author in turn trained two observers. Both observers completed the EAPRS at each school site. Ninety percent of the items had good-excellent reliability and/or high percent agreement values [22]. Any item or scale with a reliability value below 0.7 was not included in this analysis.

Using the data collected from the EAPRS tool, 14 summary exposure variables were considered. Two of the variables represented counts of the features. **Total unique types of play equipment **was a count of the following 10 features: presence of things to hang from, things to slide down, things to climb up/through, things to stand/walk on, swings, climbers/spin on, blacktop game, spring toy, imaginary play structure, and play panel. This variable ranged from 0, indicating none of these features were present, to 10, indicating the playground had all these features (see Table 1 for variable distribution information). **Total number of play features **was a summation of the total number of each of the features mentioned above with multiple credit when a playground had more than one feature (i.e., 3 slides). This variable could range from 0-∞.

**Table 1:** Descriptive characteristics of playground features, schools, and neighborhoods

	Schoolyards (N = 20)
**Playground features (possible score range)**	**Mean (SD) or %**
Total unique types of play equipment (0-10)	5.20 (1.5)
Number of play features (0-∞)	30.35 (18.26)
Overall condition (1-3)	2.50 (0.48)
Overall cleanliness (1-3)	2.67 (0.40)
Overall quality (0-1)	0.59 (0.23)
Overall safety (0-1)	0.79 (0.26)
Presence of benches, % (0-1)	30%
Presence of trash cans, % (0-1)	30%
Coverage/shade for resting features (0-3)	0.55 (1.0)
Renovated, % (0-1)	50%
**School characteristics**	
Average size of schoolyards, (sq. ft)	133,902 (34981)
Total school enrollment	442.55 (108.49)
Percentage of African-American students in school	66.0%
Population of children ≤17 in census tract	647.15 (385.80)
Percent of children ≤17 in census tract living in poverty	38.9%
**Neighborhood characteristics**	
Temperature, (degrees Fahrenheit)	73.85 (12.12)
Number of parks/green spaces in .75 mile network buffer	3.45 (1.61)
Number of free recreational centers & pools in .75 mile network buffer	0.60 (.88)
Number of commercial PA resources in .75 mile network buffer	0.30 (.57)
Street connectivity (alpha index) in .75 mile network buffer	0.40 (0.02)

Four of the variables represented the condition or safety of the features. **Overall condition **was developed by taking the mean condition of up to nine playground features (the condition of play panels is not included in the EAPRS). Condition assessed the functionality as well as the quality of the features including the presence of dents, sharp edges, rust, damage, holes, cracks, and ditches of the features. Condition ratings varied from 1 to 3 where 1 indicates poor condition and 3 indicates excellent condition. **Overall cleanliness **was developed by taking the mean cleanliness of up to 6 playground features (cleanliness of things to hang from, play panel, blacktop, and spring toy is not included in the EAPRS). Cleanliness took into consideration the presence of dirt, debris, trash, and paint quality of the features. Cleanliness ratings varied from 1 to 3 where 1 indicates not at all clean and 3 indicates mostly to extremely clean.

**Overall quality **and **overall safety **were created based on proposed variables in the EAPRS scoring sheet. To create these variables, certain attributes of each feature were dichotomously coded and the mean of these attributes was calculated. For quality, attributes considered for any type of equipment usually included condition, cleanliness, presence of 'special features', and appropriate draining features if applicable. For example, to rate the quality of things to stand or walk on, condition and cleanliness were given one point if they were rated as 3 and given 0 points if they were rated 1 or 2. A point was given if there was a bridge (a special feature); otherwise it was coded as zero. The score for the quality of things to stand or walk on was calculated as the mean of condition (0,1), cleanliness (0,1) and presence of a bridge (0,1). For swings, a point was given if condition and cleanliness were rated as 3, one point if there was proper ground drainage and 1 point if there was more than one swing type (in the absence of these features it was scored a 0). The quality of the swings was a mean of these four attributes. The overall quality of the playground was developed by taking mean quality score for all 9 features (play panels were not rated) and it ranged from 0-1. The safety variable was similarly constructed with various attributes of features coded dichotomously and averaged. Then the mean safety values across each of the nine features were averaged. Safety attributes generally included height off the ground and having a soft landing or railings as appropriate. This variable ranged from 0-1.

Three additional variables described the amenities including **presence of benches and presence of trash cans**, which were both rated as yes or no as well as **coverage/shade for resting features**, which ranged from 1-3 with 1 being poor coverage and 3 being excellent coverage. Finally, five additional characteristics were coded but were not deemed sufficiently reliable. These included: presence of any lighting at play structures or blacktops; shade/coverage over the general play structure; presence of trash; a summary graffiti variable; and the presence of rules/regulatory signs.

### Outcomes

Direct observation of utilization of the schoolyard and physical activity levels of those at the playground was completed using SOPLAY. This system involves scanning specific areas from left to right at a rate of about 1 person per second and noting characteristics of the users within the area. Characteristics documented in our study included gender, age group (adult versus child), and activity level. Participant's activity levels were classified into one of three groups: sedentary, walking (moderately active) or vigorously active. Separate scans were completed for women, men, boys and girls, so that activity levels *within *each of these categories could be considered. Ultimately all adults were combined regardless of gender because of low utilization by adults.

Schoolyards were divided into target areas that supported uniform activities and could be viewed entirely from one vantage point. The maximum number of target areas at a school was 6 and the average was 3.75. Each schoolyard was visited 10 times for one hour and thirty minutes. At each of the 10 observation sessions at a school, each target area was observed 9 times. Using momentary-time sampling, each target area was scanned for women by observer one and for men by observer two, followed by a second scan for girls by observer one and boys by observer two. Over the course of a ten minute period, each target area would be visited once. This process was completed a total of nine times per target area per observation session. The nine scans of each group (adults, girls and boys) were averaged to provide an average number of attendees during a scan for that group in a target area for that observation session. In total, our study considers 200 observations for adults, girls and boys across the 20 schools (10 observations per school). For the utilization analyses, the average number of persons across *all *target areas was examined. Hence, the 200 observations are an average of all 9 scans within an observation period and are averaged across all target areas. For the physical activity level analyses, the average of the 9 scans in the *playground target area only *were examined. Hence, the 200 observations are an average of the 9 scans of the playground target area only within an observation period.

Training for the assessors was provided and all assessors were required to meet reliability criteria [20]. Training sessions included both classroom lectures taught by the creator of SOPLAY and practice field observations. During the classroom lectures, assessors were shown how to utilize each section of the code sheet and how to discriminate between physical activity levels. Prerecorded video tapes of adolescents being physically active were also used for coding practice during the classroom lectures. In addition, practice field observations were completed over a week. During the study, additional reliability assessments were completed at 15% of the observation periods by having a third assessor complete an independent assessment. Reliabilities were found to be acceptably high (ICC = 0.71-0.97) [20]. Eleven trained assessors conducted all observations. There were two raters at each of the 200 observations (not including reliability assessments where there were three raters). The mean number of observation sessions per assessor was 36; the range was 20-47 observation sessions.

The outcome for the utilization analyses was utilization of the schoolyard, regardless of target area. Utilization describes the average number of persons per scan in any target area at the schoolyard at each observation session. Utilization by adults, girls and boys are considered separately. The outcome for the physical activity level analyses was the proportion active. For any participant at the schoolyards in the target area that contained the play set, the proportion of those who were either moderately or vigorously active was calculated by summing those in the walking or vigorous category and dividing by the total number of persons in the play set target area regardless of activity level. The proportion active for adults, girls and boys are examined separately. The rationale for limiting the target area in the physical activity level analyses to the playground target area only is that the features of the playground seemed unlikely to affect physical activity levels elsewhere on the schoolyard though the features of the playground were theorized to potentially have an effect on one's decision to come to the schoolyard, which is why all target areas were included for the utilization analyses.

### Covariates

All analyses considered 11 covariates: 4 measures representing school demographics and the surrounding area; the square footage of the schoolyard; 4 neighborhood availability/accessibility measures; the temperature at the time of the first scan for an observation session; and renovation status of schoolyard (yes/no). The 4 variables representing the demographics of the school and the surrounding area included: 1) the enrollment in the school; 2) the percentage of school students that are African American; 3) the population of children 17 and under in the census tract of the school; and 4) the percentage of children 17 and under living in poverty in the census tract of the school. Schools that were renovated as part of the "Schoolyards as Community Parks" program were designated as renovated [20]. Ten individually matched schoolyards were designated as unrenovated schoolyards. The 4 neighborhood availability/accessibility measures included: 1) a count of parks and 2) a count of the number of city run recreation centers and pools within a .75 mile network distance of schoolyards (both based on data from the city's Planning Department); 3) a count of the number of child-friendly commercial venues for activity within a .75 mile network distance of schoolyards (based on data from InfoUSA and Internet yellow pages); and 4) a measure of street connectivity (i.e., alpha index, which measures the number of circuits or loops relative to number of possible circuits or loops using a .75 mile distance ) [23]. Variable distribution information for the school and neighborhood covariates are presented in table 1.

### Statistical Analysis

All analyses were performed using Stata/IC 11.0 (StataCorp, College Station, TX). All regression models accounted for the clustering by school. Descriptive analyses were performed to examine the means and standard deviations of the different playground, school, and the surrounding neighborhood attributes as well as the average number of persons and their levels of activity.

For the utilization analyses, four different count models were considered including the negative binomial model, the poisson model, the zero-inflated poisson model, and the zero-inflated negative binomial model. Model residuals suggested negative binomial regression models be used. Given the small number of observations (200), smaller number of schools (20) and the large number of covariates (11), unadjusted negative binomial regressions were performed to determine which of the 11 covariates were significantly associated with any of the 3 utilization outcomes. For parsimonious models, only those covariates with significant associations for any group were controlled for in the primary analyses examining the associations between playground attributes and utilization.

Therefore in the utilization analyses, the primary analyses were multivariable negative binomial regressions, which adjusted for the school clustering, that predicted the average number of persons at the schoolyard during a scan as a function of each of the 9 playground attributes separately while controlling for the following significant covariates: a count of parks within a .75 mile network distance of schoolyards, the population of children 17 and under in the census tract of the school, and the enrollment in the school. Formal tests of interaction between each playground attribute and renovation status were performed for all 3 utilization outcomes (adults, girls, and boys) to examine effect modification by renovation status. Stratified analyses by renovation status were performed because the majority of the interaction tests were significant. Lastly, one negative binomial regression with all the significant playground attributes found from the primary multivariable negative binomial regression analyses was completed for both the renovated schoolyards and the unrenovated schoolyards while still controlling for the significant school/neighborhood attributes.

For the physical activity level analyses, three different outcome transformations were considered including log transformation, reciprocal transformation and untransformed. Analyses suggested that for boys and girls an untransformed proportion active outcome was the best fit. In adults, the log transformation of the proportion active outcome was the better fit. Separate unadjusted linear regressions were performed to determine which of the 11 covariates were significantly associated with each of the 3 proportion active outcomes (log transformed in adults only). For parsimonious models, only those covariates with significant associations were controlled for in the primary analyses examining the associations between playground attributes and proportion active.

Therefore in the physical activity level analyses, the primary analyses were multivariable linear regressions, which adjusted for the school clustering, that predicted the proportion moderately or vigorously active in the target area that contained the play set as a function of each of the 9 playground attributes separately while controlling for the following significant covariates: the population of children 17 and under in the census tract of the school; total number of free recreational centers and pools within a 0.75 network mile of the school; a count of parks within a .75 mile network distance of schoolyards; and renovation status. Formal tests of interaction between each playground attribute and renovation status were performed for all 3 proportion active outcomes (adults, girls, and boys) to examine effect modification by renovation status. No significant interactions were found therefore results were not stratified by renovation status but controlled for renovation status. Lastly, one multivariable linear regression with all the significant playground attributes found from the primary multivariable linear regression analyses was completed while still controlling for the significant school/neighborhood attributes. Since at least one person has to be in the playground target area to calculate the proportion active, the sample sizes for this outcome varies by group. For adults, girls and boys, the sample sizes were 61, 87, 99, respectively, meaning for 139, 113 and 101 observations there were no adults, boys or girls respectively in the playground target area.

The incident rate ratios (IRRs) and 95% confidence intervals (CIs) were reported from the negative binomial regression models and the beta coefficients and 95% CIs were reported from the linear regression models. Statistically significant results, defined as *P *≤ 0.05, are discussed.

## Results

On average, there were 5 different types of play equipment and 30 total play features (Table 1). In addition, on average the playgrounds had good-to-excellent scores for overall condition, cleanliness, quality, and safety, and 30% of the playgrounds had benches and trash cans present. At any given scan, there was an average of 2.5 persons observed at the schoolyards. Specifically, there was on average 0.4 adults, 0.9 girls and 1.2 boys (see Table 2). Overall, 52% of the persons observed on the playgrounds were moderately or vigorously active with more boys than girls classified as active (60% vs. 49%) and adults the least active (40%).

**Table 2:** Average number of persons per scan and proportion moderately to vigorously active across twenty schoolyards

	Number of observations	Number of observations with 0 persons	Mean (SD)
Avg. # of persons			
Adults	200	139	0.40 (1.10)
Girls	200	113	0.90 (2.41)
Boys	200	101	1.24 (2.86)
			
Proportion MVPA			
Adults	61	--	0.40 (0.41)
Girls	87	--	0.49 (0.33)
Boys	99	--	0.60 (0.34)

### Playground Attributes and Utilization

The IRRs for the average number of persons at the schoolyard by playground attribute for the renovated and unrenovated schoolyards are shown in Table 3. The total number of play features was positively associated with utilization at the renovated schoolyards across all groups (Table 3). Overall cleanliness was negatively associated with utilization of the renovated schoolyards in girls and boys. In addition, coverage/shade for resting features was positively associated with utilization of the renovated schoolyards in adults and boys.

**Table 3:** Clustered negative binomial regressions* between playground attributes and number of persons by renovation status

	Utilization, IRRs (95% CI)					
	
	Renovated (N = 100)			Unrenovated (N = 100)		
		
Playground Attributes	Adults	Girls	Boys	Adults	Girls	Boys
Total unique types of play equipment	1.31 (0.60, 2.87)	0.74 (0.30, 1.81)	0.81 (0.44, 1.49)	0.74 (0.53, 1.02)	1.01 (0.70, 1.46)	1.06 (0.79, 1.43)
Total # of play features	**1.08 (1.07, 1.09)**	**1.07 (1.05, 1.09)**	**1.05 (1.03, 1.06)**	0.99 (0.91, 1.07)	1.00 (0.94, 1.06)	1.01 (0.95, 1.07)
Overall condition	18.22 (0.66, 499.60)	22.64 (0.89, 575.46)	3.85 (0.50, 29.50)	0.58 (0.31, 1.08)	0.65 (0.33, 1.29)	0.83 (0.41, 1.68)
Overall cleanliness	0.74 (0.24, 2.29)	**0.24 (0.09, 0.65)**	**0.54 (0.29, 0.99)**	1.38 (0.51, 3.75)	0.94 (0.40, 2.22)	**0.51 (0.30, 0.87)**
Overall quality	0.32 (0.02, 4.60)	0.19 (0.005, 7.71)	0.18 (0.02, 1.47)	0.36 (0.04, 3.10)	**0.22 (0.06, 0.79)**	0.43 (0.17, 1.06)
Overall safety	0.56 (0.01, 48.42)	18.73 (0.23, 1498.0)	2.74 (0.20, 37.28)	0.41 (0.12, 1.44)	5.33 (0.63, 45.35)	**3.77 (1.51, 9.40)**
Presence of benches	3.64 (0.79, 16.67)	1.81 (0.30, 10.85)	2.08 (0.66, 6.61)	0.41 (0.04, 4.19)	**0.26 (0.07, 0.94)**	0.28 (0.08, 1.04)
Presence of trash cans	3.31 (0.81, 13.50)	2.62 (0.59, 11.71)	2.23 (0.88, 5.68)	0.34 (0.10, 1.09)	1.11 (0.55, 2.22)	**1.80 (1.16, 2.77)**
Coverage/shade for resting features	**2.89 (1.79, 4.64)**	1.80 (0.93, 3.48)	**1.83 (1.21, 2.76)**	0.62 (0.26, 1.48)	0.75 (0.35, 1.58)	0.82 (0.45, 1.49)

Significant results (p <.05) represented in bold.

*Adjusted for a count of parks within a .75 mile network distance of schoolyards, the population of children 17 and under in the census tract of the school, and the enrollment in the school.

IRR = Incident Rate Ratio. CI = Confidence Interval.

Overall quality and presence of benches were negatively associated with utilization at the unrenovated schoolyards in girls (Table 3). Overall safety and presence of trash cans was positively associated with utilization in boys while overall cleanliness was negatively associated with utilization of unrenovated schoolyards in boys.

The IRRs for the average number of persons at the schoolyard are shown in Table 4 from analyses that simultaneously consider all the significant playground attributes for the renovated schoolyards and unrenovated schoolyards. After controlling for the other significant playground attributes and the significant school/neighborhood attributes, the total number of play features remained positively associated with utilization at the renovated schoolyards in adults and girls. The association became marginally significant in boys, *P *= .08 (Table 4). The IRRs were either the same or only slightly attenuated. Overall cleanliness remained negatively associated with utilization of the renovated schoolyards in the same groups (e.g., girls and boys) though the IRRs were somewhat attenuated. In addition, coverage/shade for resting features remained positively associated with utilization of the renovated schoolyards in the same groups (e.g., boys and adults). The IRRs decreased in magnitude for adults but not boys.

**Table 4:** Combined clustered negative binomial regressions* between playground attribute and number of persons by renovation status

	Utilization, IRRs (95% CI)					
	
	Renovated (N = 100)			Unrenovated (N = 100)		
		
Playground Attributes	Adults	Girls	Boys	Adults	Girls	Boys
Total # of play features	**1.07 (1.06, 1.07)**	**1.07 (1.06, 1.08)**	1.02 (1.00, 1.03)	--	--	--
Overall cleanliness	--	**0.29 (0.15, 0.54)**	**0.27 (0.13, 0.56)**	**--**	**--**	1.56 (.52, 4.64)
Overall quality	--	**--**	**--**	**--**	0.26 (0.02, 4.58)	--
Overall safety	--	**--**	**--**	**--**	**--**	**2.77 (1.05, 7.32)**
Presence of benches	--	**--**	**--**	**--**	0.82 (0.06, 11.22)	**--**
Presence of trash cans	--	**--**	**--**	**--**	**--**	2.02 (0.88, 4.63)
Coverage/shade for resting features	**1.44 (1.29, 1.61)**	--	**2.03 (1.40, 2.92)**	**--**	**--**	**--**

Significant results (p <.05) represented in bold.

*Additionally adjusted for the significant playground attributes, a count of parks within a .75 mile network distance of schoolyards, the population of children 17 and under in the census tract of the school, and the enrollment in the school.

IRR = Incident Rate Ratio. CI = Confidence Interval.

The overall quality, cleanliness and presence of trash cans were no longer significantly associated with utilization of the unrenovated schoolyards after controlling for the other significant playground attributes and the significant school/neighborhood attributes (Table 4). Overall safety remained significantly and positively associated with utilization of the unrenovated schoolyards in boys.

### Playground Attributes and Physical Activity Levels

The beta coefficients for the proportion active (MVPA) by playground attribute are shown in Table 5. Significant, positive associations were observed for the presence of benches and coverage/shade for resting features in boys and a significant, negative association was observed for overall safety in boys. The beta coefficients for the proportion active (MVPA) in boys when simultaneously considering all the significant playground attributes in the subsequent regression analyses are shown in Table 6. After including all the three significant playground attributes together with the significant school/neighborhood attributes, all associations were attenuated and no longer significant.

**Table 5:** Clustered linear regressions* between the proportion moderately to vigorously active and playground attribute

	Proportion Active (MVPA), Beta Coefficients (95% CI)		
	
Playground Attributes	Adults N = 61	Girls N = 87	Boys N = 99
Total unique types of play equipment	-0.04 (-0.15, 0.07)	-0.002 (-0.088, 0.084)	0.07 (-0.01, 0.14)
Total # of play features	0.008 (-0.000, 0.000)	0.003 (-0.003, 0.009)	0.001 (-0.005, 0.007)
Overall condition	0.23 (-0.13, 0.58)	0.07 (-0.04, 0.18)	0.07 (-0.12, 0.26)
Overall cleanliness	-0.04 (-0.31, 0.24)	-0.05 (-0.20, 0.09)	0.05 (-0.15, 0.25)
Overall quality	-0.003 (-0.672, 0.665)	0.13 (-0.12, 0.37)	0.12 (-0.26, 0.49)
Overall safety	0.36 (-0.53, 1.26)	0.21 (-0.30, 0.72)	**-0.48 (-0.88, -0.07)**
Presence of benches	0.16 (-0.15, 0.47)	-0.03 (-0.18, 0.11)	**0.12 (0.01, 0.24)**
Presence of trash cans	0.10 (-0.12, 0.32)	-0.01 (-0.12, 0.10)	-0.02 (-0.15, 0.11)
Coverage/shade for resting features	0.06 (-0.06, 0.18)	0.02 (-0.05, 0.10)	**0.07 (0.01, 0.12)**

Significant results (p <.05) represented in bold.

*Adjusted for the population of children 17 and under in the census tract of the school, total number of free recreational centers & pools within a .75 mile network distance of schoolyards, count of parks within a .75 mile network distance of schoolyards and schoolyard renovation status

MVPA = Moderate-to-Vigorous Physical Activity. CI = Confidence Interval.

**Table 6:** Combined clustered linear regressions* between the proportion active (MVPA) and playground attribute

	Proportion Active (MVPA), Beta Coefficients (95% CI)
	
Playground Attributes	Boys, N = 99
Overall safety	-0.30 (-0.97, 0.37)
Presence of benches	0.004 (-0.267, 0.275)
Coverage/shade for resting features	0.04 (-0.07, 0.16)

Significant results (p < .05) represented in bold.

* Adjusted for the for the significant playground attributes, population of children 17 and under in the census tract of the school, total number of free recreational centers & pools within a .75 mile network distance of schoolyards, count of parks within a .75 mile network distance of schoolyards and schoolyard renovation status

MVPA = Moderate-to-Vigorous Physical Activity. CI = Confidence Interval.

## Discussion

This study examined the association of specific playground features with utilization of the schoolyards and physical activity levels of those on the playground. This is one of the first studies to use direct observation and a detailed park assessment to examine this association. Only a few playground attributes were significantly associated with utilization in at least one group (e.g., adults, girls, or boys). Fewer playground attributes were significantly associated with proportion active and these were found only in boys and were no longer significant in analyses that controlled for all significant playground attributes.

In the multivariable analysis for the renovated schoolyards, significantly greater utilization was associated with total number of play features in adults and girls; in boys it was marginally significant. Specifically, for a one unit increase in the number of play features, the predicted counts would increase by a factor of 1.08 for adults, 1.07 for girls and 1.05 for boys. For instance, if there were 10 additional play features, the rate of boys using the schoolyard would be 50% higher. This finding is consistent with another study that used the EAPRS measurement tool and found the availability of more features (e.g., trails, wooded area, playground, base diamond, basketball court), in particular trails, was associated with adult utilization of parks for physical activity [12].

Our study also found that coverage or shade for resting features was significantly associated with greater utilization. Boys utilized the schoolyards at a rate of nearly 2 times more when there was moderate coverage relative to poor coverage over the resting features in analyses that controlled for all significant variables; for adults the rate of utilization was almost 50% higher. Shade has been named previously as an important feature of parks by parents in a qualitative study conducted in Canada [24]. Another study found that step counts were higher at preschool outdoor environments that featured trees and shrubbery [25]. Hence the availability of shade across various features is likely an important amenity for parks and playgrounds. The results of this study also suggested that overall cleanliness was significantly associated with *less *utilization of the renovated playgrounds. This finding may simply be because a playground may be less clean when there are a large number of persons utilizing it. At a minimum, it is likely to be relatively clean if no one is using it. The only other study that examined information on condition and cleanliness of the features and amenities at a park using a park assessment could not examine their effects due to a lack of variability [12].

Compared to the renovated schoolyards, unrenovated schoolyards had fewer significant associations between utilization and playground attributes and after further control for all the significant playground attributes, only one variable remained significant: overall safety remained significantly associated with utilization in boys. At schoolyards with the highest levels of safety across all playground features (i.e., scored a perfect 1), rates of utilization were nearly 3 times as high relative to schoolyards that scored the lowest on safety across all playground features (i.e., scored a 0). Safety was highly correlated with both total number of unique types of play equipment and total number of play features (.65 and .63, respectively) as well as with condition (r = .46) see Table 7. The unrenovated playgrounds had wide variability in several exposure variables and it may be that a minimum threshold of features or conditions must be met before there is significant utilization (although all playgrounds in the study did have functional playgrounds). Future studies with greater sample sizes and more power may be able to elucidate these relationships across playgrounds that have large variability. It may be found that the same attributes, namely a large number of play features, is a salient factor for all playgrounds regardless of renovation status when a minimum standard is met.

**Table 7:** Correlation of playground attributes with school and neighborhood attributes

	Overall condition	Total unique types of play equipment	Total number of play features	Overall cleanliness	Overall quality	Overall safety	Presence of benches	Presence of trash cans	Coverage/shade for resting features
**Overall condition**	1.00								
**Total unique types of play equipment**	0.58*	1.00							
**Total # of play features**	0.43	0.56*	1.00						
**Overall cleanliness**	-0.09	-0.21	-0.13	1.00					
**Overall quality**	0.71*	0.11	0.14	0.34	1.00				
**Overall safety**	0.46*	0.65*	0.63*	-0.43	-0.08	1.00			
**Presence of benches**	0.37	0.28	0.02	0.21	0.20	-0.08	1.00		
**Presence of trash cans**	0.00	0.28	0.23	-0.40	-0.37	0.22	0.29	1.00	
**Resting features coverage/shade**	0.28	0.27	0.01	0.06	0.09	-0.05	0.86*	0.30	1.00
**Average size of schoolyards**	-0.17	-0.20	0.05	-0.01	-0.15	-0.33	0.09	0.07	-0.05
**Number of parks/green spaces**	-0.01	-0.29	-0.13	-0.06	0.11	-0.18	-0.40	-0.19	-0.56*
**Number of recreational centers & pools**	-0.01	0.49*	0.01	-0.27	-0.16	0.15	-0.08	0.05	0.02
**Number of commercially available PA resources**	-0.31	-0.07	-0.28	-0.40	-0.38	-0.13	0.04	0.63*	0.06
**Connectivity**	-0.11	0.03	0.02	-0.16	-0.22	-0.12	0.08	0.14	-0.02
**Population of children ≤17**	-0.16	-0.29	0.00	0.13	-0.08	0.09	-0.05	-0.08	-0.17
**Total school enrollment**	-0.08	-0.01	0.08	-0.22	-0.17	0.15	-0.31	0.04	-0.35
**Percentage of children **≤**17 living in poverty**	0.05	-0.19	-0.20	-0.23	0.20	-0.16	-0.36	-0.22	-0.36
**Percentage of African-American students**	-0.16	0.11	-0.23	-0.24	-0.15	0.04	-0.17	-0.08	-0.08
**Temperature**	-0.03	0.35	0.22	-0.16	-0.29	0.37	0.16	0.40	0.37

* Significant results (p <.05) represented in bold with an asterisk

Very few associations were found between the playground attributes and the proportion active and after further control for all the significant playground attributes, no associations remained significant. This suggests that features and amenities may draw people to the playground but once there, these features do not seem to influence the level of activity. This is similar to earlier findings where there was more utilization at renovated playgrounds compared to unrenovated playgrounds but little difference in activity levels [20]. Future research is needed to understand what features and amenities can increase activity levels of those who use parks and playgrounds.

This study is not without limitations. First, there were only 20 schoolyards assessed in this study which makes it difficult to tease out any correlated effects. Second, the clustered negative binomial regressions resulted in a few extremely large 95% confidence intervals for a couple playground attributes. This is most likely due to the small sample of schoolyards and a small sample of persons observed utilizing specific schoolyards. The small sample size may have also resulted in limited power to detect differences, in particular for the analyses that considered physical activity levels. Thirdly, SOPLAY was used to collect physical activity levels of adults even though the original intent of this instrument is to collect activity levels in youth. However, a similar direct observation protocol (SOPARC) has been validated in adults [26]. Finally due to the large number of analyses performed, some significant findings may be due to chance, though overall the patterns of findings seem to be consistent across age and gender.

A strength of this study was the use of direct observation to assess utilization and physical activity levels at the schoolyards. This study was able to accurately capture real-time data on what took place at specific schoolyards. The majority of studies to date have simply examined whether features are available in the park and physical activity levels overall rather than utilization of the specific park or activity at the park. In addition, a detailed environmental assessment method was utilized to rate and score a variety of different playground attributes rather than just noting the presence of the feature. This type of detailed research has been called for but rarely implemented [27,9].

More research is needed to further understand how to combine the large number of descriptive variables that results from environmental audits. This study used several variables suggested from the EAPRS scoring sheet and alternative specifications may have resulted in different findings. To confirm these findings, additional studies that examine these associations at a larger number of sites are needed. In addition, examining these associations utilizing direct observation and a detailed environmental assessment method not only in schoolyards, but in other public physical activity resources such as parks and other playgrounds would be informative.

## Conclusions

Our findings indicate that a small number of specific playground attributes, most notably total number of play features, are associated with utilization of renovated schoolyards. Activity levels of those at the playground were similar regardless of features or amenities. Information from this study can be used to create more user-friendly physical activity spaces, which may help to address the obesity epidemic. Future research is needed to corroborate these findings and explore whether any different features or amenities influence the levels of activity in those using these facilities.

## Competing interests

The authors declare that they have no competing interests.

## Authors' contributions

NC conceived and designed the study. All authors made substantial contributions to the analysis and interpretation of data. All authors were involved in drafting the manuscript and have given final approval of the version to be published.
